# Small-Pox in Buffalo

**Published:** 1888-08

**Authors:** 


### SMALL-POX IN BUFFALO.

There has been during the past month a few cases of small-pox in Buffalo, and the people were at one time greatly alarmed, fearing that the disease would become epidemic. The first case appeared in the person of John Jackson, a colored man. The disease proved fatal within twenty-four hours after discovery. The house was quarantined and the inmates and neighbors vaccinated. No new cases have developed in the neighborhood.

The following week nine new cases were discovered within two days among the Poles of the Sixth Ward. Two of the patients had attended school up to the time the eruption appeared on the body. There were 1,800 children in this school, not half of whom had been vaccinated, and the appearance of cases in five different houses led the Health Department to fear a serious spread of the disease. The patients were all removed to the Quarantine Hospital, with the exception of one on Townsend street, occupying an isolated house, which could be easily quarantined.

The infected bedding was destroyed, the house thoroughly fumigated, the inmates rigidly quarantined, and 16,000 people living in the district were vaccinated. No new cases were discovered until ten days after, when two were discovered on Detroit street, where cases had already developed. On the following day two cases were discovered nearly half a mile east of Detroit street, and another on Townsend street. One of the patients had not been vaccinated, and had a fatal form of the disease; the other exposed at the same time had been vaccinated after exposure and six days previous to the appearance of the eruption. As a result, the disease in the latter patient is of a mild type. All of the twelve Polish cases in the Sixth Ward are relatives, and the origin of the disease has been traced to a man who came from a town in Prussia, Poland, where small-pox was epidemic. He came to live with a family on Townsend street, bringing some infected clothing with him. In this house the first case was developed, and as there was no physician in attendance, the disease was not discovered until others were affected.

Our active and intelligent Health Physician, Dr. Edward Clark, cannot be too highly complimented for the earnestness with which he engaged in the work, eradicating the disease from the city. A Bureau of Vaccination was established in the Polish district, and the entire medical staff of the Health Department, sixteen physicians in all, were kept at work until nearly all the inhabitants living in that portion of the city bounded by William, Smith, Sycamore and Rother streets, were vaccinated. The Polish people, with very few exceptions, assisted the physicians in their efforts to enforce general vaccination, several thousands coming to the office, where physicians were in attendance day and night. Had it not been for the thoroughness with which the Health Department did its work, a serious epidemic would undoubtedly have taken place, for, in no other part of Buffalo are the conditions more favorable for the spread of any contagious disease.

The patients at the Quarantine Hospital have done remarkably well. Of the thirteen patients, there has been but one death, and that was a child with hemorrhagic variola. Four patients with confluent variola are convalescent, although the case-book of the hospital shows that cases of confluent small-pox have hitherto terminated fatally. We believe that these excellent results are due to an innovation in the management of the Quarantine Hospital proposed by Dr. Clark. A resident physician, Dr. C. B. LeVan, a young gentleman of exceptional ability, was appointed to give the patients his constant attention. The wisdom of making such an appointment is shown by the results, a large proportion of recoveries, the amount of disfiguration from the disease, which has been greatly reduced, and the dread which patients have hitherto had of going to a suburban hospital with no physician in constant attendance has been in a great measure removed.

---

IN EXAMINING the library of our late editorial *confrère*, Dr. Davidson, several valuable books are missing, and the executor is anxious to have them returned. Among the books which have been loaned to his professional friends and not returned, are: Allan's Commercial and Organic Analysis; first volume of Ashurst's International Encyclopædia of Surgery; seventeenth volume Popular Science Monthly. This editorial notice is intended for our city readers, and if any have the above-mentioned works, they will materially assist the family of our deceased brother by returning them at once. Dr. Davidson's library is also offered for sale, and physicians will find it to their advantage to call upon Mrs. Davidson for any books they may need.

